# Inhibitory control training in healthy and highly educated older adults

**DOI:** 10.1590/1980-57642021dn15-030012

**Published:** 2021

**Authors:** Corina Satler, Edison Tostes Faria, Gabriel Neiva Rabelo, Ana Garcia, Maria Clotilde Henriques Tavares

**Affiliations:** 1 Faculdade de Ceilândia, Universidade de Brasília – Brasília, DF, Brazil.; 2 Laboratory of Neuroscience and Behaviour, Department of Physiological Science, Institute of Biology, Universidade de Brasília – Brasília, DF, Brazil.

**Keywords:** aging, cognition, executive function, quality of life, neuropsychology, idoso, cognição, função executiva, qualidade de vida, neuropsicologia

## Abstract

Executive function training is considered a promising tool for delaying the natural effects of aging on cognition. However, there are still few studies that propose a unimodal intervention with a focus on inhibitory control, and none of them has studied the effect of this type of intervention on older adults (OA).

**Objective:**

The aim of this study is to investigate the benefits of inhibitory control training in healthy OA by comparing the two assessment time points, namely, before and after training.

**Methods:**

Twenty-seven participants were included after interview and checking the inclusion criteria. The training was based on the stop-signal paradigm and carried out in 21 sessions.

**Results:**

Participants performed better after training by reducing the false alarm error rate (i.e., for stop-signal trials), reducing omission error rate, showing an increase in hit rate, Go response time (i.e., for go-signal trials), stop-signal response time, and showing a decrease in the level of anxiety. The executive function training had no significant impact on the scores obtained in the complementary neuropsychological tests.

**Conclusions:**

These results are consistent with previous studies that support the viability and effectiveness of cognitive intervention for executive functions in OA and suggest a positive effect of the intervention, which may be related to the learning experience of a new and challenging task.

## RESUMO.

O treinamento das funções executivas é considerado uma ferramenta promissora para retardar os efeitos naturais do envelhecimento na cognição. No entanto, ainda são poucos os estudos que propõem uma intervenção unimodal com foco no controle inibitório, e nenhum deles estudou o efeito desse tipo de intervenção em idosos.

**Objetivo:**

Investigar os benefícios do treinamento de controle inibitório em idosos saudáveis, comparando os dois momentos da avaliação: antes e depois do treinamento. **Método:** Um total de 27 participantes foi incluído após entrevista e verificação dos critérios de inclusão. O treinamento foi baseado no paradigma *Stop-Signal* e realizado em 21 sessões.

**Resultados:**

Os participantes tiveram um melhor desempenho após o treinamento, reduzindo a taxa de erro de alarme falso (nas tentativas *stop*), reduzindo a taxa de omissões e mostrando aumento na taxa de acerto e GoTR (nas tentativas *go*), no SSRT e mostrando uma diminuição significativa no nível de ansiedade. O treinamento não teve impacto significativo nas pontuações obtidas nos testes neuropsicológicos complementares.

**Conclusões:**

Esses resultados são consistentes com estudos anteriores que sustentam a viabilidade e eficácia da intervenção cognitiva para funções executivas em idosos, além de sugerirem um efeito positivo da intervenção, que pode estar relacionado à experiência de aprendizagem de uma tarefa nova e desafiadora.

## INTRODUCTION

Aging is a worldwide demographic phenomenon, practically all societies currently experience an increase in the population of adults over the age of 60 years.^1^ There is a broad consensus that aging is associated with changes in neurobiological functions at various levels; for example, reduced gray matter volume and changes in various regions of the cortex which are crucial for higher cognitive functions, such as the prefrontal cortex (PFC), medial, and parietal cortex,^2^ ^,^ ^3^ and changes in white matter connectivity between the prefrontal and posterior cortical regions and within the posterior sensory cortices.^4^ These age-related changes are associated with a slight impairment in several cognitive domains, including episodic and operational memory and attentional and inhibitory processes.^5^ ^-^ ^7^

Among cognitive domains, executive functions (EFs) play a crucial role in the successful completion of complex tasks in everyday life.^8^ In general, they are associated with social, occupational adaptation, and the physical and mental health of individuals.^5^ EFs correspond to a set of cognitive skills that facilitate the appropriate execution of behaviors aimed at goals, which are important in the face of new or ambiguous situations that require adjustment, adaptation, or flexibility of behavior to the demands of the environment.^9^ ^,^ ^10^

Executive functions include three core components, namely, working memory, cognitive flexibility, and inhibitory control. The latter consists of the ability to control attention, behavior, thoughts, and emotions as it acts as a brake on automatic behavior allowing generating an appropriate response. It involves two fundamental components, namely, self-control (i.e., behavioral inhibition) and interference control (i.e., selective attention and cognitive inhibition).^5^ Self-control can be defined as the ability to interrupt (abruptly) a planned and continuous thought or action.^11^ In numerous situations in real life, planned or ongoing actions are suddenly rendered inappropriate by unforeseen events or changes in the immediate environment,^12^ and it is necessary to inhibit habitual behavior to make new, adaptive, and more flexible choices.^5^

In the benign aging process, EFs are considered an indicator of active aging and longevity, and there is evidence that they can be improved with training and practice.^5^ Thus, maintaining the performance of executive functioning at high levels is associated with success in managing the activities of daily living and social skills, contributing to the promotion of health, functional independence, autonomy of older adults (OA), and improvement of their quality of life.^13^

In this sense, cognitive interventions have shown promise in healthy aging^13^ ^,^ ^14^ and in elderly people.^15^ Such interventions are based on the premise that the brain, even with age, maintains neuroplasticity, that is, the ability to adapt or benefit from experiences, thoughts, and emotions,^16^ resulting in structural behavioral and brain changes at the cellular level.^17^ There are three main cognitive intervention techniques, namely, cognitive rehabilitation, cognitive stimulation, and cognitive training. The latter is characterized by the practice of standardized activities in order to maintain or improve the functioning of cognitive functions.^18^ Cognitive training can vary in relation to periodicity, format, cognitive skills targeted for intervention (i.e., multi domain or unimodal), type of instruments, method of administration (i.e., pencil and paper or computerized tasks), focus on the effect of the practice on the target skills, and on maintaining the effects of the intervention over time.^19^

Computerized cognitive training (CCT) is characterized by the use of mobile electronic devices, such as a computer, laptop, or tablet.^16^ The review and meta-analysis studies have shown the effectiveness of CCT in improving the general cognitive performance of healthy OA, with specific benefits in episodic memory, attention, working memory, processing speed, visuospatial skills, and EFs,^20^ ^,^ ^21^ and also the sustainability of the effects over time, the ability to transfer to untrained cognitive domains, and the generalization of the effects on daily functioning.^8^

Despite the relevant role of EFs in everyday life, there is a lack of studies on cognitive interventions focusing on these processes, when compared with other cognitive domains, such as memory.^13^ ^,^ ^18^ Within the perspective of EF’s CCT studies, most of them are multimodal interventions, in which EF training is the only one aspect within a larger program.^8^ ^,^ ^21^ Among the studies that propose a unimodal intervention, the focus has been especially on training working memory.^5^ ^,^ ^22^ There are few studies of inhibitory control,^23^ ^-^ ^25^ and none of them involved aging population. Developing cognitive interventions focusing on inhibitory processes is of crucial importance because there is a visible loss of this function during healthy cognitive aging.^5^ Thus, the objective of this study is to investigate whether OA benefit from the performance of an inhibitory control training (ICT) by comparing the two assessment time points, namely, before and after training. We expected a better performance in the trained cognitive ability after training, with an increase in hit rate for go-signal trials and a decrease in the false alarm error rate for stop-signal trials.

## METHODS

All subjects were volunteers and signed an informed consent document in accordance with the Ethical Guidelines for Research with Human Subjects (196/96 and 251/97 CNS/MS Resolution). This study was approved by the Human Subjects Ethics Committee of the Health Sciences Faculty of the University of Brasilia (protocol 36747614.5.0000.0030).

In this study, 27 healthy OA (>60 years old) were participated and recruited from the community. All were native Brazilian Portuguese speaker with normal or corrected-to-normal vision and hearing. They were right-handed volunteers (i.e., Edinburgh Laterality Inventory),^26^ with no history of neurological or psychiatric episodes and no recent use of psychotropic medication, as evaluated by a detailed anamnesis. All participants scored at least 24 on the Montreal Cognitive Assessment (MoCA), according to the years of education (≥12),^27^ less than 13 points on the Beck’s Depression Inventory (BDI-II),^28^ and less than 7 points on the Beck’s Anxiety Inventory (BAI).^29^

### Instruments

**Figure 1.**
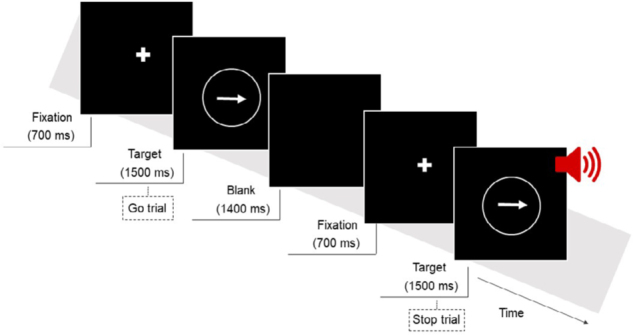
Experimental procedure of the stop-signal task. The task consisted of Go and Stop trials. All trials began with a central fixation cross and were followed by an arrow. Participants were required to press a key (i.e., either left or right depending on the orientation of the arrow). On 25% of the trials, an auditory containment signal would appear as a signal to withhold response.

A computerized version of the “stop-signal task” (SST) paradigm,^30^ adapted in our laboratory, was used to perform the ICT. Participants were instructed to respond as fast as possible to the initiation signal (i.e., go signal) by pressing a key (i.e., either left or right depending on the orientation of the arrow). They were warned that in a minority of trials, an auditory containment signal (i.e., stop signal) would be presented and should inhibit the previously planned response. A pilot study was conducted with the aim of adjusting the temporal parameters to verify the understanding of the task instructions and to increase the probability of completing the task successfully. The task consists of 128 trials, of which, 25% are stop signals, totaling 32 chances to inhibit the action. The tasks starts with a fixation cross displayed in the center of a computer screen (17") for 700 ms, followed by an arrow pointing left or right (with a frequency of relative appearance 1:1), which serves as an initiation signal (i.e., go signal), presented for 1,500 ms, and then, a blank screen for 1,400 ms (see Figure 1). In 25% of the tests, an auditory containment signal of 500 Hz (i.e., stop signal) is played after the go signal and displayed for 500 ms, with 50 ms of adjustment as a latency variable known as the inhibitory signal delay [stop-signal delay (SSD)]. The analysis included as follows: hit rate, omission error rate, Go response time (GoRT) for go-signal trials, false alarm error rate for stop-signal trials, and the stop-signal response time (SSRT).

Two neuropsychological tests extensively used for both experimental and clinical purposes in the assessment of OA^31^ were applied before and after ICT as follows: (1) Semantic Verbal Fluency (SVF) test: it measures EF and language.^32^ Animals and fruits versions were used. The total number of words spoken in 1 minute, excluding repetitions and errors, as well as the performance as a function of time (intervals of 0–15, 16–30, 31–45, and 46–60 s), were analyzed. (2) Stroop test: it assesses aspects of EF, sustained and selective attention.^33^ Subjects are required to verbalize the printed color for each stimulus presented as fast as possible. A computerized version of the Victoria Stroop task adapted in our laboratory was used. Moreover, an alternative version of the Victoria Stroop task was used, maintaining the same sequence, amount of stimuli, and neutral words as the Victoria version. The rate of self-correction, errors, omissions, and reaction time (RT) in each condition was recorded for each participant. After a pilot study, no differences were found on performance in both versions of the task.

The five questions that were answered by the participants (i.e., yes/no) were elaborated for this study as follows: Q1. The cognitive training up to your expectations?; Q2. Do you think that the cognitive training was useful in your daily life?; Q3. Did you notice any benefits in your way of acting during training?; Q4. Did you notice any benefit in concentration during a simple activity, for example: reading a book, or during a complex activity, for example: driving during training?, and Q5. Would you recommend this training to others?

### Procedure

The application of instruments and cognitive intervention was carried out individually and took place in a room with lighting and noise control. Each participant was scheduled during the morning or afternoon according to their convenience and availability and carried out all phases of the study. This methodological criterion was incorporated to control the changes in the performance of the participants due to the variation in the time of testing, which is more pronounced in aging populations.^34^

The study was carried out in three phases, namely, baseline, ICT (i.e., began 1–2 days following the phase 1 and took place across 21 sessions with a frequency of three times a week), and post-training (which was scheduled at an interval of 7 days from the final training session). The entire collection takes 9 weeks. To perform both EF tests avoiding learning effect,^34^ participants were randomly subdivided into two subgroups (i.e., A and B). Thus, in phase 1, after performing the SST task, subgroup A performed the SVF-Animals and Stroop-Alternative tests, and subgroup B performed the SVF-Fruits and Stroop-Victoria tests. In phase 3, after performing the SST task, subgroup A performed the SVF-Fruits and Stroop-Victoria tests, and subgroup B performed the SVF-Animals and Stroop-Alternative tests. At the end of phase 3, the BAI and BDI-II inventories were reapplied and participants answered five self-report questions about the training and possible benefits.

During each ICT session, participants performed the SST and filled out a daily form in which they were consulted about the quality of sleep from the previous night, food, mood, feelings, unusual events, use of new medication, beginning of sports practice, or leisure activities.

### Statistical analysis

To characterize the sample regarding the demographic variables of interest, descriptive analyzes were implemented, using mean and standard deviation. The independent samples *t*-test was used to compare the demographic and mental status of participants in subgroups A and B. To analyze the cognitive training data, Wilcoxon’s test was conducted using the IBM *Statistical Package for the Social Sciences* (SPSS) software (v.25.0 for Windows). The dependent variables for ICT were false alarm error rate, hit rate, omission error rate, GoRT, and SSRT, and there were 17 variables from the two neuropsychological tests. The independent variable was the assessment time point. The level of statistical significance was set at 5% (p<0.05) for all tests.

## RESULTS

The 27 OA had a mean age of 69.22 years (SD: 0.89), mean education of 16.75 years (SD: 0.79), and were 70% female. Subgroups (A and B) of participants did not differ for age, years of schooling, and mental status (MoCA). The demographic data and scores on cognitive screening test are shown in Table 1.

**Table 1.** Demographic and scores on cognitive screening of total participants and subgroups A and B.

		Total sample	Subgroups		p-value
			A	B	
		(n=27)	(n=13)	(n=14)	
Gender (%)	Female	70	62	79	–
	Male	30	38	21	–
Age – Mean (SD)		69.22 (0.89)	67.69 (1.06)	70.64 (1.33)	0.201
Education (years) – Mean (SD)		16.75 (0.79)	17.30 (1.05)	16.25 (1.18)	0.592
MoCA – Mean (SD)		26.92 (0.24)	27.15 (0.33)	26.71 (0.36)	0.473

MoCA: Montreal Cognitive Assessment.

The analysis of differences in SST task’s performance before and after training for each variable using the Wilcoxon test (Table 2) revealed a statistically significant difference between the two assessment time points. Results showed improved post-training performance for stop-signal trials (false alarm error rate: Wilcoxon, z=-4.21, p<0.001), go-signal trials (hit rate: Wilcoxon, z=-4.34, p<0.001, omission error rate: Wilcoxon, z=-4.34, p<0.001, and GoRT: Wilcoxon, z=-2.85, p=0.004), and SSRT (Wilcoxon, z=-3.27, p<0.001).

**Table 2.** Inhibitory control performance before and after training.

Variable	Assessment time point		p-value
	Before	After	
	Mean (SD)	Mean (SD)	
Go-signal trials – Hit rate (%)	86.45 (13.75)	97.22 (3.11)	0.001*
Go-signal trials – Omission error rate (%)	13.54 (2.64)	2.54 (0.59)	0.001*
Go response time – GoRT (ms)	598.61 (19.74)	686.88 (20.28)	0.004*
Stop-signal trials – False alarm errors (%)	43.75 (2.95)	18.63 (2.70)	0.001*
SSTR (ms)	176.72 (18.35)	226.98 (14.17)	0.001*

Wilcoxon test. *p<0.05.

Regarding psychological scores in pre- and post-training evaluations, a Wilcoxon test for paired samples revealed for BAI measures (baseline=3.77±2.13; post-training=2.96±2.73), absence of a statistically significant difference between pre- and post-training sessions (Wilcoxon, z=-1.76, p=0.078), as well as no significant difference (Wilcoxon, z=-0.60, p=0.548) for BDI-II inventory (baseline=6.29±4.24; post-training=5.85±4.04).

Table 3 shows cognitive outcome variables. By using a Wilcoxon test for paired samples, it was revealed for Stroop task measures no significant difference between the two assessment time points. In the SVF, there were statistically significant differences in the pre- and post-training sessions for total words (i.e., subgroups A, p<0.001 and B, p<0.001), and the following variables: SVF (0–15 s) for subgroup A (p<0.005), SVF (15–30 s) for subgroups A (p=0.008) and B (p=0.014), and SVF (45–60 s) for subgroup B (p=0.025).

**Table 3.** Executive performance before and after inhibitory control training by subgroups A and B.

	A			B		
	Assessment time point		p-value	Assessment time point		p-value
	Before	After		Before	After	
	Mean (SD)	Mean (SD)		Mean (SD)	Mean (SD)	
Stroop 1 (self-correction)	0.14 (0.53)	0.08 (0.28)	0.655	0.79 (1.18)	0.62 (0.96)	0.435
Stroop 1 (errors)	0.00 (0.00)	0.08 (0.28)	0.317	0.00 (0.00)	0.00 (0.00)	1.000
Stroop 1 (omissions)	0.29 (0.61)	0.00 (0.00)	0.102	0.14 (0.53)	0.00 (0.00)	0.317
Stroop 1 (RT)	744.04 (126.82)	694.03 (90.53)	0.221	795.55 (219.78)	717.88 (131.20)	0.101
Stroop 2 (self-correction)	0.43 (0.64)	0.17 (0.38)	0.157	2.79 (51.36)	1.00 (1.15)	0.667
Stroop 2 (errors)	0.00 (0.00)	0.00 (0.00)	1.000	0.36 (0.84)	0.00 (0.00)	0.102
Stroop 2 (omissions)	0.00 (0.00)	0.00 (0.00)	1.000	0.00 (0.00)	0.00 (0.00)	1.000
Stroop 2 (RT)	827.12 (139.18)	718.46 (123.43)	0.087	784.01 (158.77)	797.08 (113.03)	0.972
Stroop 3 (self-correction)	2.00 (1.88)	1.33 (1.23)	0.280	2.36 (2.67)	3.08 (2.43)	0.474
Stroop 3 (errors)	0.36 (0.74)	0.00 (0.00)	0.102	0.50 (1.34)	0.62 (0.87)	0.496
Stroop 3 (omissions)	0.14 (0.53)	0.00 (0.00)	0.317	0.14 (0.36)	0.15 (0.37)	1.000
Stroop 3 (RT)	974.52 (102.54)	930.45 (114.94)	0.249	991.15 (162.49)	941.15 (162.49)	0.279
SVF (total words)	24.57 (1.73)	18.07 (1.14)	0.001*	15.30 (1.20)	20.69 (1.09)	0.001*
SVF (0–15s)	5.78 (0.47)	3.76 (0.56)	0.005*	3.75 (0.27)	4.30 (0.57)	0.359
SVF (15–30s)	4.85 (0.58)	2.69 (0.38)	0.008*	2.41 (0.52)	3.61 (0.46)	0.014*
SVF (30–45s)	3.35 (0.46)	2.46 (0.60)	0.095	1.75 (0.50)	4.07 (0.45)	0.025*
SVF (45–60s)	10.57 (0.80)	8.76 (0.64)	0.156	7.58 (0.49)	8.69 (0.72)	0.195

SVF: Semantic Verbal Fluency Test. Wilcoxon test. *p<0.05.

A descriptive analysis of the responses of daily form along training showed 93% answers “yes” to item 10, related to the quality of sleep from the previous night, followed by 40% of affirmative responses to item 7, referring to coffee consumption in the past 2 hours prior to the training session. Affirmative responses to item 11, regarding the experience of mood swings or signals of emotional exhaustion, were averaged 7% and were mainly related to a health concern with a family member (Table 4).

**Table 4.** Mean percentage of YES responses along the training sessions.

Daily form	S1	S2	S3	S4	S5	S6	S7	S8	S9	S10	S11	S12	S13	S14	S15	S16	S17	S18	S19	S20	S21
Q1. Did you start taking a new medication?	4%	4%	0%	0%	0%	0%	0%	0%	4%	0%	0%	0%	0%	0%	0%	0%	0%	0%	0%	0%	0%
Q2. Did you use any medicine to sleep or relax last night?	0%	0%	0%	0%	0%	0%	0%	0%	0%	0%	0%	0%	0%	0%	0%	0%	0%	0%	0%	0%	0%
Q3. Did you start the practice of any physical activity?	0%	0%	0%	0%	0%	0%	0%	0%	0%	0%	0%	0%	0%	4%	0%	0%	0%	0%	0%	0%	0%
Q4. Did you have started any new leisure activity?	0%	0%	0%	0%	0%	0%	0%	0%	0%	0%	7%	0%	0%	0%	7%	0%	0%	0%	0%	0%	0%
Q5. Did you drink alcohol in the past 24 hours?	0%	4%	4%	11%	0%	4%	7%	0%	4%	4%	4%	4%	0%	4%	0%	4%	0%	0%	0%	0%	0%
Q6. Did you use drugs in the past 24 hours?	0%	0%	0%	0%	0%	0%	0%	0%	0%	0%	0%	0%	0%	0%	0%	0%	0%	0%	0%	0%	0%
Q7. Did you drink coffee in the past two hours?	44%	48%	44%	41%	33%	48%	44%	33%	41%	37%	37%	41%	37%	37%	41%	37%	41%	33%	44%	30%	37%
Q8. Did you drink soda or some energy drink, or eat chocolate in the past 2 hours?	4%	0%	0%	4%	0%	7%	4%	4%	4%	0%	4%	0%	4%	0%	4%	4%	0%	4%	0%	7%	4%
Q9. Have you been experiencing any unusual events?	0%	0%	0%	0%	4%	0%	0%	0%	0%	0%	4%	0%	0%	0%	0%	0%	0%	0%	0%	7%	0%
Q10. Did you have a good night’s sleep last night?	89%	85%	89%	96%	89%	85%	93%	89%	93%	96%	96%	96%	96%	96%	93%	100%	96%	96%	96%	85%	96%
Q11. Did you have experienced mood swings or emotional distress signs?	11%	19%	7%	7%	4%	7%	7%	4%	4%	7%	7%	7%	7%	4%	11%	15%	0%	7%	4%	4%	7%

Finally, five self-report questions indicated that for 78% of the sample, the training met their expectations, bringing some benefit in their daily lives (74%), with improvement in their concentration (67%), and decreased behaviors or responses performed prematurely (33%). When we asked participants if they would recommend training to other people, 96% responded positively.

## DISCUSSION

In this study, we investigated whether OA benefit from performing an ICT by comparing the two assessment time points, namely, before and after training. General results indicated a performance improvement on the SST task, with an increased precision of responses throughout the sessions. Such findings are consonant with the study developed by Berkman et al.^23^ who found better performance over the sessions in a sample of university students using the same paradigm. Additionally, the results fit the literature report on CCT, in which a positive impact on target skills is observed through training practices.^13^ ^,^ ^14^

Inhibitory control is commonly studied using paradigms, such as SST, which is based on the premise that motor acts can be planned and suspended before their execution. Logan and Cowan^30^ proposed that performance in the task is modeled by a “horse race model” between parallel and independent processes of initiation and containment, with SSRT being a way of measuring the latency of the control act generated internally.

Results obtained in this study indicated a significant difference in the SSRT between the two assessment time points, with higher values after training, and higher values in GoRT. Such results are in accordance with indications in the literature of the adoption of strategies by the participants aiming at precision in performance and, consequently, neglecting the speed in the execution of the task.^35^ Thus, the anticipation of a containment signal would result in a permanent braking process, which manifests itself with the slowest RT. Another explanatory hypothesis is about the difficulty of the task, assuming that a short SSD facilitates the act of canceling the action; meanwhile, a long SSD turns it more difficult. However, the values obtained in the SSRT fall within the time estimates published in the literature (from 150 to 300 ms, with an average of 200 ms).^30^

Regarding the neuroanatomical substrate of the inhibitory control, studies indicate that it is mediated by the frontal lobes, specifically the lateral and dorsomedial PFC, with an interaction between frontal, posterior, and subcortical connections aimed at processing the stimulus.^23^ A meta-analysis study developed by Swick et al.^35^ showed that two dissociable neural systems contribute to the effectiveness of the response inhibition and the relevance of the system depends on the nature of the task. One system is the cingulo-opercular network, including the anterior PFC, anterior insula, the anterior dorsal cingulate cortex, and thalamus; the other is the frontoparietal network, including the dorsolateral PFC and intraparietal groove. During the execution of the SST, greater activation of the lateral and medial PFC has been reported, close to the parietal cortex with greater dominance of the right hemisphere. Greater activation of the bilateral anterior and medial insular cortex BA 6 (SMA/pre-SMA) has been correlated with good performance and success on the task.^23^ ^,^ ^35^

In the context of aging, inhibitory mechanisms are compromised, possibly due to the gray matter volume reduction and changes in various regions of the cortex, such as the PFC, medial, and parietal temporal cortex,^2^ ^,^ ^3^ and the dysfunction of connectivity between anterior and posterior areas.^4^ Concomitantly, studies have documented evidence that the decreasing speed on cognitive tasks due to aging is associated with the loss of white matter.^12^ ^,^ ^36^ However, the improvement in participants’ performance after the ICT suggests a positive effect of the intervention, which may be related to the learning experience of a new and challenging task. Berkman et al.^23^ reported an adaptive change in the functional cortical cerebral organization through functional neuroimaging after performing the ICT. In this sense, we can assume that brain changes related to neuroplasticity could also be observed in OA taking into account our behavioral results and assuming that brain plasticity is evident throughout adulthood.^7^

In the learning context, the transfer from a trained to an untrained task occurs when these two tasks share processing components and activate overlapping brain regions.^37^ The Stroop test is one of the most used measures of inhibitory control^34^ and has been related to the probability of contention in the SST.^38^ However, our results did not reveal significant differences in RT between the two assessment time points. As expected, low errors and omissions rates were observed, considering that sample profile.

For SFV test-total words, superior results were observed for Animals before and after training, suggesting that it is a less complex task. Participants obtained lower word generation in the first 15 s of the tests and higher word production in the 45- to 60-s interval in both categories (i.e., animals and fruits) caught our attention. This particularity in performance is opposed to that is described in the literature on OA.^32^ ^,^ ^38^ A possible explanation is the presence of psychological factors, such as anxiety about the performance of the test.^34^

In terms of mood, the levels of self-reported anxiety (BAI) and depression (BAI) remained stable, with values below the cutoff level.

Regarding self-report questions, results suggest that the ICT could have generalizing effects, contributing to the participants’ daily lives, for example, in concentration during a simple activity, such as reading a book and impulsiveness. It should be noted that although similar results have been reported in other studies, suggesting the benefit of cognitive intervention for everyday situations and functional activities,^8^ the results obtained in this study must be taken with caution because subjective measures can be contaminated with bias from the informant, affecting its validity.

Regarding the cognitive training format (i.e., individual sessions), there was a strong adherence to the proposal by the participants. This characteristic of the sample is of great relevance, suggesting the presence of a strong motivational factor, which favors the engagement in activities and is generally observed in studies using the collective intervention modality.^13^ ^,^ ^19^

In general, the results of this study are consistent with previous studies that support the viability and effectiveness of EF intervention in OA and that inhibitory control performance can be maintained and even improved through a cognitive intervention. For future studies, the methodological replication of this study is suggested, including a larger sample, participants with different levels of education, structured scales for assessing motivational aspects and daily life, neuroimaging studies, and conducting a longitudinal follow-up to verify the effects of the intervention’s durability.

This study has the following limitations: (1) the sample size and female prevalence in the sample composition; (2) the sample evaluated had a very high level of education, which does not represent the reality of most OA in Brazil; (3) the nonassessment of language comprehension; (4) the nonuse of structured scales for assessing motivational and everyday life aspects; (5) the noninclusion of a control group in the study design; and (6) the maintenance of the long-term performance improvement was not investigated.
